# A systematic review on the effects of endometriosis on sexuality and couple’s relationship

**Published:** 2020-10-08

**Authors:** P Norinho, MM Martins, H Ferreira

**Affiliations:** Centre for Psychology at University of Porto, Centro Materno-Infantil do Norte – Centro Hospitalar do Porto, Porto, Portugal; Centre for Psychology at University of Porto, Faculty of Psychology and Education Science, University of Porto, Porto, Portugal; Abel Salazar Institute of Biomedical Sciences, University of Porto, Portugal, Centro Materno- Infantil do Norte – Centro Hospitalar do Porto, Porto, Portugal.

**Keywords:** Endometriosis, sexuality, couple

## Abstract

**Background:**

Endometriosis is likely to affect sexuality and intimate relationships but the effect endometriosis has on partners remains overlooked and the existing studies show conflicting results. The effect of the disease and its treatment on the couple may be pronounced given the absence of an obvious cause or cure, the likelihood of chronic recurring symptoms, and the potential impact on both sex and fertility.

**Materials and methods:**

We followed the PRISMA guidelines to conduct this systematic review, which involved a database search of published available research related to the effects of endometriosis treatment on sexual function, couple’s relationship and on the partner published between 2000 and 2020.

**Results:**

The studies considered revealed that women with endometriosis report a significant effect of the disease on sexuality and relationship. Also, most of the published studies suggest that the impact on partners may be profound, affecting many life domains including sex, intimacy and the relationship in general.

**Conclusions:**

Data suggests that male partners should not be overlooked in the treatment of endometriosis and that psychosocial support including sexual and couple therapy might be beneficial.

## Introduction

Endometriosis is a benign chronic inflammatory and oestrogen dependent disease, defined by the presence of endometrial gland and stroma-like tissue outside of the uterus (Ballard et al., 2008; Berek et al., 2012). It is one of the most common gynaecological diseases. The exact prevalence of endometriosis is unknown, but estimates indicate that it affects around 10% of women of reproductive age and around 30- 50% of women with infertility and/or pelvic pain (Signorello et al., 1997). The diagnosis is based on the woman’s history, symptoms and signs. It is corroborated by physical examination and imaging techniques and finally proven by histology of either a directly biopsied vaginal lesion, from a scar, or of tissue collected during laparoscopy (Dunselman et al., 2014).

The most common symptoms are progressive dysmenorrhoea, deep dyspareunia, chronic pelvic pain unrelated to the menstrual cycle, and infertility (Sinaii et al., 2008). Dyspareunia is a complaint of 32-70% of women with endometriosis (De Graff et al., 2013). Nevertheless, the presence of pain at penetration is not the only determinant of these women’s sexual health (Melis et al., 2015). Studies on the sexual function of women with chronic diseases showed that these affect multiple domains of sexual function (Rosen et al., 2006). Endometriosis can have a significant effect on various aspects of women’s lives, including their social and sexual relationships, work, and study (De Graaff et al., 2013).

In addition to the classic symptoms, women with endometriosis are more likely to develop depression and anxiety (Lorençatto et al., 2006; Pope et al., 2015) and their quality of life may be affected by pain, the emotional impact of sub-fertility, a possible recurrence of the disease and uncertainty about the future related to repeated surgeries and the long duration of medical therapy (Berek et al., 2012).

Furthermore, chronic illnesses like endometriosis are likely to affect patients’ partners. The effect of endometriosis on partners and the couple dynamic could be especially pronounced given the absence of an obvious cause or cure, the likelihood of chronic, recurring symptoms and the potential impact on both sex and fertility (Dunselman et al., 2014).

A number of studies in women have evaluated the negative effect of the disease on sexual function (Ferrero et al., 2005; Fritzer et al., 2013; De Graaff et al., 2016) and the effect of surgery and hormonal treatment on deep dyspareunia. However, the extension and duration of the positive effects of treatment are not well defined and overall sexual function and the quality of the relationship remain under-investigated. Studies on the partners and the relationship are scarce, and given the growing evidence on the importance of the partner in the process of coping with other chronic illnesses there is a need to focus on the impact of endometriosis on partners and the relationship.

The need to investigate the psychosocial impact of endometriosis on women and partners was highlighted in the 2013 ESHRE Guidelines on The Management of Women with Endometriosis (European Society of Human Reproduction and Embryology, 2013). Here we aim to shed some light on sexuality and the couple’s relationship, including the partner’s perspective on the impact of the disease, an aspect usually overlooked.

The aim of this article is to review the current body of knowledge on the impact of endometriosis on the couple’s relationship and male partners. To our knowledge, this is the first systematic review on endometriosis that takes into account the perceptions of both the relationship and sexual function perceptions, the partners’ perspective and that includes both qualitative and quantitative studies.

## Materials and methods

This systematic review was conducted according to the Preferred Reporting Items for Systematic Reviews and Meta-Analyses (PRISMA)guidelines (MoherET AL., 2009).

We searched the electronic databases PubMed and MEDLINE to identify English language, peer-reviewed journal articles published between January 2000 and December 2020 on the effects of the disease and of its treatment on sexual function, the couple’s relationship and on the partner. The following combination of MeSH terms was used: (‘endometriosis’) AND (‘dyspareunia’ OR ‘sexual function’ OR ‘sexuality’ OR ‘sexual dysfunction’) AND (‘relationship’ OR ‘couple’ OR ‘marital adjustment’). All relevant articles were examined, and their reference lists were systematically reviewed to identify other studies for potential inclusion in this review.

## Results

**Figure 1:**
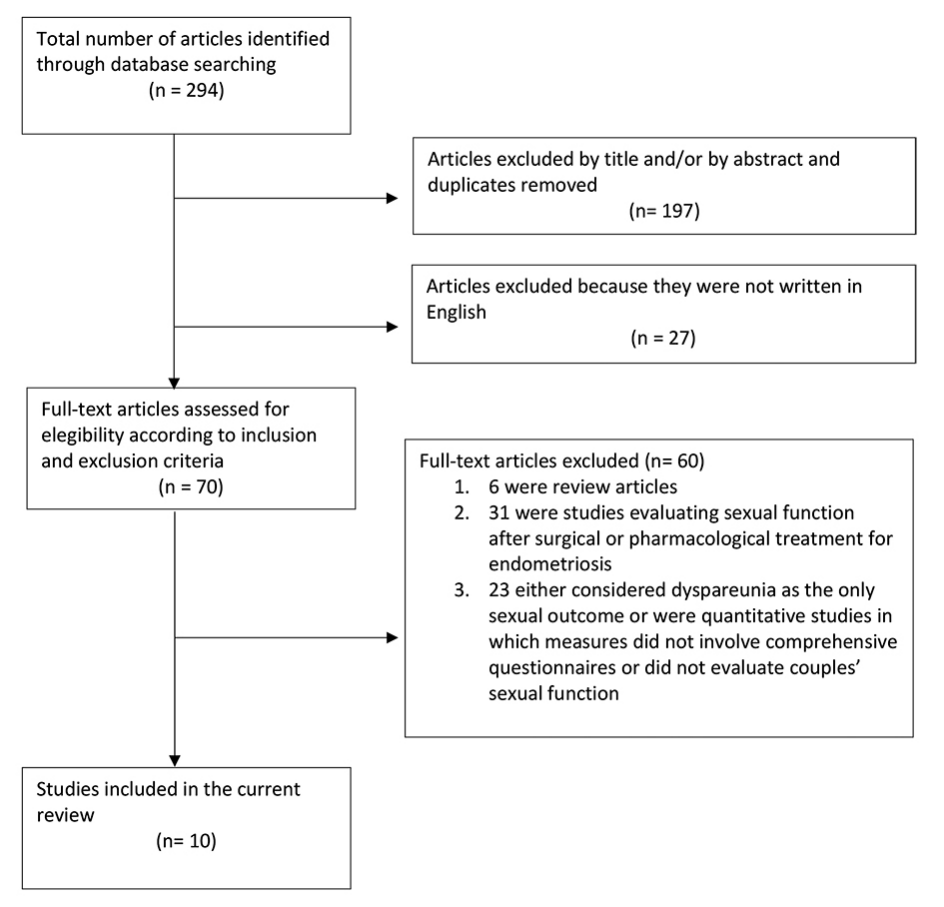
Flow diagram of the literature search.

The flow diagram is depicted in Figure 1. A total of 294 articles were identified as potentially relevant. Of these, 197 were excluded by title and/ or by abstract or were duplicates removed and 27 were excluded for being written in a language other than English. The remaining 70 had titles or abstracts reporting information on the association between endometriosis and sexual functioning and were thus retrieved for evaluation. Of these, 6 were narrative review articles, 31 were studies evaluating sexual function after surgical or pharmacological treatment of endometriosis, and 23 either considered dyspareunia as the only sexual outcome or were quantitative studies in which measures did not involve comprehensive questionnaires or did not evaluate couples’ sexual function. The final selection included ten studies. The studies were selected due to the fact that they were studies that focused on the impact of endometriosis on both sexual function and couple’s relationship.

Table I summarizes results regarding the characteristics of the selected studies (author, year, country, study, aims, study design, sample, type of endometriosis, sexual functioning measures). Overall, these studies used data from 582 women with endometriosis and 411 partners. Sample sizes varied between 13 (Butt and Chesla, 2007) and 170 (Ferrero et al., 2005) women, and between 13 (Butt and Chesla, 2007) and 236 (Hammerli et al., 2018) men. Two studies involved female controls (De Graff et al., 2016; Hammerli et al., 2018), one involved woman with gynaecological conditions other than endometriosis (Ferrero et al., 2005), and one involved male partners of healthy women (De Graff et al., 2016). The type of endometriosis was specified in 3 studies (Ferrero et al., 2005; Fritzer et al., 2013; Hammerli et al., 2018), two of them considered patients score stage I to IV (Fritzer et al., 2013; Hammerli et al., 2018) and one divided the patients in two groups (a group of patients with Deep Infiltrating Endometriosis (DIE) of uterosacral ligaments and another group with endometriosis without lesions of the uterosacral ligaments) (Ferrero et al., 2005).

**Table I:** Characteristics of studies included in systematic review of the effects of endometriosis treatment on sexual function and couple’s elationship.

Author Year	Country	Study aims	Study design	Sample	Type of Endometriosis	Measures and methods
Ferrero et al., 2005	Italy	To characterize sexual functioning among women with endometriosis and deep dyspareunia	Case-control	96 women with endometriosis (76 with USLE and 20 without USLE) and 40 controls (women with infertility, pelvic pain, ovarian cysts, and uterine leiomyomas)	Two groups of patients with endometriosis: one group with DIE of the uterosacral ligament and another without uterosacral lesions.	Sexual Satisfaction Subscale of the DSFI; GSSI; VAS
Fernandez et al., 2006	Australia	To explore the experiences of partners of women with endometriosis	Qualitative	16 male partners completed questionnaires pertaining to the impact of endometriosis; 3 male partners participated in additional semi structured interviews.	Type of endometriosis of the female partner not specified	Thematic analysis of combined questionnaire and interview data
Denny and Man, 2007	UK	To determine how much of an impact endometriosis associated dyspareunia has on the lives and relationships of women	Qualitative (story-telling approach and semi structed interviews)	30 women with endometriosis	Not specified	Semi-structured interviews
Butt and Chesla, 2007	USA	To investigate the responses in couples’ relation- ships to living with chronic pelvic pain from endometriosis	Qualitative, interviews	13 women with endometriosis and 13 male partners of women with endometriosis	Not specified	Thematic analysis of interview data
Fagervold et al., 2009	Norway	To investigate longitudinally the consequences of endometriosis in women diagnosed with the disease 15 years ago	Qualitative, interviews	78 women with endometriosis	Not specified	Author devised questionnaire
Fritzer et al., 2013	Austria and Germany	To evaluate the prevalence and the impact of sexual dysfunction, sexual distress, and inter-personal relationships in patients with endometriosis	Multicentre cohort study	125 women with endometriosis	Women with rAFS stage I to IV	FSFI, FSDS, FSDS, NAS
De Graff et al., 2016	The Netherlands	To compare sexual functioning between women with endometriosis and a control group and to compare sexual functioning of male partners of women with endometriosis and male partners of women in the control group	Cross-sectional study	83 women with endometriosis and 40 women without endo- metriosis; 73 male partners of women with endometriosis and 26 male partners of women without endometriosis	Not specified	Women: FSFI, SF-12, PCS, SSCS, HADS, VAS and author devised questionnaire Men: IIEF and author devised questionnaire
Culley et al, 2017	UK	To investigate the impact of endometriosis on male partners of women with the condition	Cross-sectional qualitative study	22 women with endometriosis and 22 male partners of women with endometriosis	Not specified	Semi-structured interviews
Ameratunga et al, 2017	Australia	To determine how endometriosis affects the quality of life of partners of women with endometriosis and how it impacts their relationship, finances, mental state and daily living	Cohort study questionnaire- based	51 partners of women with endometriosis	Not specified	Questionnaires based on the UK Endopart Study (Culley et al, 2017)
Hammerli et al, 2018	Switzerland, Germany, Austria	To investigate how male partners experience sexuality in partnership with women with endometriosis	Multi-centre case control study	236 partners of endometriosis patients and 236 partners of age matched control women without endometriosis with a similar ethnic background	Women with ASRM score stage I to IV	BISF, SHF and author devised questionnaire

Abbreviations: ASRM: American Society for Reproductive Medicine; BISF: Brief Index of Sexual Functioning; DSFI: Sexual Satisfaction Subscale of the Derogatis Sexual Functioning Inventory; FSDS: Female Sexual Distress Scale; FSFI: Female Sexual Function Index; GSSI: Global Sexual Satisfaction Index; NAS: Numeric Analogue Scale; rAFS: revised American Fertility Society; SHF: Sexual History Form; USLE: Deep infiltrating endometriosis of the uterosacral ligament; VAS: visual analogue scale.

Of the 10 included studies, five used a qualitative methodology (Culley et al., 2017; Fernandez et al., 2006; Denny and Man, 2007; Butt and Chesla, 2007; Fagervold et al., 2009) and five were quantitative studies. All qualitative studies used semi-structured interviews as assessment except for one, which also used a story telling approach (Denny and Man, 2007). Among the quantitative studies there was one cross-sectional (De Graff et al., 2016), one case-control (Ferrero et al., 2005), one multicentre cohort (Fritzer et al., 2013), one single centre cohort (Amertuga et al., 2017) and one multi-centre case-control (Hammerly et al., 2018). The sexual questionnaires used were the Sexual Satisfaction Subscale of the Derogatis Sexual Functioning Inventory (DSFI) and the Global Sexual Satisfaction Index (GSSI) (Ferrero et al., 2005), Female Sexual Function Index (FSFI) and SSCS (De Graff et al., 2016), Brief Index of Sexual Functioning (BISF) and Sexual History Form (SHF) (Hammeli et al., 2018).

The main results of the studies are shown in Table II. Ferrero et al. investigated 96 patients with endometriosis and 40 patients without diagnosed endometriosis but with infertility, pelvic pain, ovarian cysts, and uterine leiomyomas. The main objective of this quantitative study was to characterise the sexual function in women with endometriosis and deep dyspareunia. No significant differences were observed between patients with and without endometriosis regarding satisfaction with the partner, variety in sex life, and interest in sex. Still, communication about sexuality was perceived as significantly worse for endometriosis patients than for those without the disease (Ferrero et al., 2005). However, other studies led to different conclusions pointing endometriosis as a cause of problems in the relationship and relationship break up and a cause of sexual problems or sexual dysfunction (Denny and Man, 2007; Butt and Chesla, 2007; Fagervold et al., 2009; Fritzer et al., 2013). Dyspareunia was pointed as a cause of sexual dysfunction in 4 studies (Denny and Man, 2007; Fagervold et al., 2009; Fritzer et al., 2013; De Graff et al., 2016), with two of them reporting an association between endometriosis and an avoidance of intercourse (Denny and Man, 2007; Fritzer et al., 2013). One study points out that interventions to enable couples to address the impact of endometriosis on sexual relations are limited and those that are available are reported by couples to be unhelpful (Butt and Chesla, 2007). Partners are considered the main source of support to endometriosis patients’ in a stable relationship (Denny and Man, 2007; Butt and Chesla, 2007).

**Table II:** Main results of studies included in systematic review of the effects of endometriosis treatment on sexual function and couple’s relationship.

Author Year	Results
Ferrero et al., 2005	No significant difference between women with DIE of the uterosacral ligament (group U), endometriosis without infiltration of the uterosacral ligament (group E) and controls (group C) in the satisfaction with the partner, variety in sex life, and interest in sex. The communication about sex was significantly worse in groups U and E when compared with group C (P<.05).
Fernandez et al., 2006	Male partners of women with endometriosis with low mood, anxiety and powerlessness that contributed to a grief process. Some reported acceptance and relationship growth.
Denny and Man, 2007	Women with endometriosis described their partners as supportive but a number spoke of the tensions and arguments caused by the lack of sexual relations. Partners were reported as feeling rejected by the lack of sexual activity and younger women in particular felt that the lack of sexual activity jeopardised the relationship, some women reported that a relationship had broken up because of dyspareunia and the avoidance of sexual intercourse.
Butt and Chesla, 2007	The experience of living with endometriosis disrupted day-to-day life and intimate relatedness for couples. Five relationship coping patterns: ‘together but alone’, ‘battling together’, ‘conjoined through disability’, ‘totalized by caregiving’ and ‘engaged in mutual care’. Interventions to enable couples to address the impact of endometriosis on sexual relations are limited and those that are available are reported by couples to be unhelpful.
Fagervold et al., 2009	Symptoms of endometriosis caused problems in their relationship in 15.4% and broken relationship in 7.7%; 48.7% reported that endometriosis had caused problems with their sex life. Significant correlation between dyspareunia and a negative influence on relationship (p=.004).
Fritzer et al., 2013	Female sexual distress in 78% and female sexual dysfunction in 32% of women with endometriosis. A statistically significant correlation between sexual dysfunction and pain intensity during intercourse, lower number of episodes of sexual intercourse per month, greater feelings of guilt toward the partner, and fewer feelings of femininity.
De Graff et al., 2016	Women with endometriosis, when compared with controls, reported significantly more dyspareunia (53% vs15%, P<.001) and more impairment in global sexual functioning (assessed with FSFI questionnaire). No differences were found between the sexual function (IIEF questionnaire) of male partners of women with and without endometriosis. Logistic regression analysis indicated that dyspareunia and depressive symptoms were independent and significant negative predictors for sexual functioning.
Culley et al, 2017	Male partners reported that endometriosis affected many life domains including sex and intimacy, planning for having children, working lives and household income. It required them to take on additional support tasks and roles having an impact on emotions creating helplessness, frustration, worry and anger. Male partners have a marginalized status in endometriosis care due to the absence of recognition of the impact on male partners and lack of support available to man.
Ameratunga et al, 2017	Male partners reported negative feelings about the diagnosis of endometriosis (92%), that endometriosis affected their day-to-day life moderately or severely (70%), felt that their finances were affected (52%), felt that sex life (74%) and their relationship as a whole (56%) were affected. The ones whose relationship had been affected by endometriosis had also more likely day to day life (P=.0027), sex life (P=.001) and finances (P=.002) affected. 80% reported receiving no informa- tion about the impact of the disease on couples and only 34% felt that health professionals had engaged them in the decision-making process and had been supportive of them.
Hammerli et al, 2018	Male partners reported that endometriosis affected sexuality (75%). When compared with controls, both groups were satisfied with their sexual relationship (73.8% vs 58.1%, P=.002) but more partners of women diagnosed with endometriosis were not satisfied (P=.002) and their sexual problems more strongly interfered with relationship happiness (P=.001). Frequencies of sexual intercourse (P<.001) and all other partnered sexual activities (oral sex, petting) were significantly higher in the control group. The wish for an increased frequency of sexual activity (P=.387) and sexual desire (P=.919) did not differ statistically between both groups).

Abbreviations: FSFI: Female Sexual Function Index; IIEF: International Index of Erectile Function.

The first study to focus on male partners was conducted by Fernandez et al. (2006). This was a qualitative study with 16 male partners that completed closed-ended questionnaires on the impact of endometriosis. Three of these participated in additional semi-structured interviews. The purpose of this study was to explore the experiences of partners of women with endometriosis. Low moods, anxiety, and powerlessness were identified and contributed to a grief-like process. Nevertheless, some also reported acceptance and relationship growth. The second study (Butt and Chesla, 2007) was also qualitative and had similar results. The main objective was to investigate how living with chronic pelvic pain from endometriosis affected couples’ relationships and had identical results. Thirteen women with endometriosis and respective partners reported disruptions to day-to-day life and a significant impact on sexuality and intimacy (Butt and Chesla, 2007).

De Graff (2016) published the first quantitative study comparing the sexual function of couples facing endometriosis to those who do not, using a cross-sectional sample of 83 women and their partners (n=74). Women with endometriosis reported significantly more frequent pain during intercourse, higher levels of chronic pain, more sexual functioning impairment, more pain catastrophising, and more depression and anxiety symptoms. On the other hand, no significant differences were found on the Index of Erectile Function between the male counterparts, the sexual function of male partners of women with endometriosis was comparable to the sexual function of the control group, based on the Index of Erectile Function suggesting that endometriosis does not affect the sexual function of male partners.

In 2017 an analysis of the ENDOPART study was published. This was a qualitative study of 22 women with endometriosis and their male partners (n=44). Contrary to the results previously published by De Graaf (2016), this study provides evidence that the impact on partners may be profound. Endometriosis affected many life domains including sex and intimacy, planning for having children, working lives and household income. Feelings of helplessness, frustration, worry and anger were also identified on male partners (Culley et al., 2017). Women reported dyspareunia, general fatigue, a reduced sexual desire as a result of medication, low moods, stress in trying to conceive, bleeding during and/or after sex and feeling unattractive and unfeminine.

Also in 2017, a semi-quantitative cohort study including 51 partners of women with endometriosis was published (Ameratunga et al., 2017). The objective was to determine how endometriosis affects their quality of life and relationships, finances, mental state and daily living. Partners reported a significant effect on their sex life (74%) and their relationship as a whole (56%).

The most recent study was a multi-centre case- control study performed between 2010 and 2015 (Hammerli et al., 2018). Partners of endometriosis patients (n=236) and the same number of partners of age-matched control women without endometriosis were asked to answer selected, relevant questions of the Brief Index of Sexual Functioning and the Global Sexual Functioning questionnaire, as well as some investigator-derived questions. Many partners reported changes in sexuality (75%) and, even though a majority of both groups was satisfied with their sexual relationship, more partners of diagnosed women were not satisfied and their sexual problems more strongly interfered with relationship happiness than partners of undiagnosed women. The frequency of sexual intercourse and all other partnered sexual activities (oral sex, petting) was significantly lower. Nevertheless, the wish for an increased frequency of sexual activity and sexual desire did not differ statistically between both groups.

Two studies report an absence of recognition of the impact of endometriosis on male partners and a lack of support available to men (Culley et al., 2017; Ameratunga et al., 2017).

## Discussion

Endometriosis seems to affect women, their partners and the relationship unit. Women with endometriosis report a significant effect of the disease on sexual function and on the relationship. The lack of communication about sexuality, sexual problems or dysfunction and avoidance of sexual intercourse are some of the aspects pointed as possible causes for problems in the relationship and even relationship break up. Dyspareunia, although a frequent complaint, is not the only determinant of the sexual function in endometriosis patients. Women living with this condition also report more pain catastrophising and more depression and anxiety symptoms that, indirectly, may have an impact on sexual function and the relationship. Male partners are generally seen as a source of support to the patient with endometriosis.

Gynaecologists that work with these patients should always inquire about the couple’s sexual health since dyspareunia is a complaint of 32-70% of women with this disease (De Graff et al., 2013) and it affects multiple domains of sexual function (Rosen et al., 2006). The circle of perpetration of sexual dysfunction in women with endometriosis commonly starts with the presence of recurrent dyspareunia, and the consequent fear and anticipation of pain. Fear and anticipation of pain are two powerful inhibitors of the sexual response cycle, and the main contributors to low desire and lubrication (Di Donato et al.,2014; Fritzer et al., 2013; Ferrero et al., 2005).

Reducing pain at penetration is therefore the first step in the treatment of women with sexual dysfunction associated with endometriosis. This can be achieved with a combination of medical and surgical therapy, the use of a vaginal lubricant (reducing the friction caused by penetration), modification of sexual technique (increasing foreplay, delaying penetration, trying alternative sexual positions). Considering that psychological dimensions and relational mechanisms also play an important role in sexual function, sexual therapy can be very useful when sexual problems cause distress and thus improve physical intimacy.

Few studies have focused on the partner’s perspective on the impact of the disease on their quality of life, sexual function and the relationship. Except for the study published by De Graaf (2016), that suggested that sexual function in male partners was not affected by endometriosis, most studies suggest that the impact on partners may be profound. In De Graaf (2016) study the majority of participants (patients and partners) were enrolled in a tertiary care centre. This means that all couples included in this study were ‘survivors’ in relationship terms and this may have underestimated the impact the disease may have in terms of sexual function and relationship.

Partners of women with endometriosis report that the disease affected many life domains including sex and intimacy and their relationship as a whole. Nevertheless, even though the frequency of sexual intercourse and all other partnered sexual activities were lower, desire did not seem to be affected. The disease had an impact on planning for having children, working lives and household income and feelings of helplessness, frustration, worry and anger were identified and these may be intensified by an absence of recognition of the impact of endometriosis on male partners and the lack of support available to man. Also, the effect of medical and surgical treatment on the partners has not been directly investigated.

In short, the published studies examined support the fact that endometriosis affects the couple’s sexual function and relationship. However, even though several studies included the assessment of sexual function and its impact on women with endometriosis, research on the impact of the disease and its treatment using the couple as the unit of analysis is lacking. In effect, both male partners’ perceptions and relationship dynamics are overlooked in the treatment of the disease. This leads to a lack of support available to men and to the couples, with most centres not offering neither sexual nor couples’ therapy. Since partners are generally considered a source of support to the patients with endometriosis, future research is needed to investigate the impact of endometriosis longitudinally using the couple as unit of analysis.

The heterogeneity (different research methods, study aims, questionnaires used, measures to evaluate sexual function) and the small number of existing studies on the partner’s perspective of the impact of the disease on their quality of life, sexual function and the relationship are two limitations of the available evidence. Another limitation is the fact that the effect of medical and surgical treatment on the partners has not been directly investigated. Nevertheless, and to our knowledge, this is the first systematic review on the current body of knowledge on the impact of endometriosis on the couple’s relationship, taking into account relationship and sexuality perceptions from both the patients and their partners and including both qualitative and quantitative studies.

## Conclusion

Endometriosis affects sexual function and the couple’s relationship, and research on the impact of the disease and its treatment using the couple as a unit of analysis is lacking. Women with endometriosis report a significant effect of the disease on sexual function and the romantic relationship and most of the published studies suggested that the impact on partners may be profound, affecting many life domains including sex, intimacy and the relationship in general.

Even though few studies have focused on the partners, the disease seems to have a big impact on the partner’s sexual function and quality of life. Since male partners and the relationship are overlooked in the treatment of the disease there is a lack of support available to men (and couples as a whole) and most of the centres do not offer sexual or couple’s therapy.

Partners are generally considered a source of support to the patients with endometriosis and future research is needed to investigate ways to address the male partner and the relationship as a whole. Data suggests that male partners should not be overlooked in the treatment of endometriosis and that psychosocial support including sexual and couple therapy might be beneficial.
